# Prevalence and patterns of use of mantra, mindfulness and spiritual meditation among adults in the United States

**DOI:** 10.1186/s12906-017-1827-8

**Published:** 2017-06-15

**Authors:** Adam Burke, Chun Nok Lam, Barbara Stussman, Hui Yang

**Affiliations:** 1 Institute for Holistic Health Studies, Department of Health Education/HSS327, San Francisco State University, 1600 Holloway Avenue, San Francisco, California, 94132 USA; 2 Department of Preventive Medicine, Health Behavior Research Program, University of Southern California, 2001 N Soto Street, Los Angeles, CA 90032 USA; 3 National Center for Complementary and Integrative Health (NCCIH), National Institutes of Health, 6707 Democracy Boulevard/Suite 401, Bethesda, MD 20892 USA; 4 Department of Computer Science, San Francisco State University, 1600 Holloway Avenue, San Francisco, California, 94132 USA

**Keywords:** Meditation, National Health Interview Survey (NHIS), Health promotion, Mind body therapies, Complementary therapies, Integrative medicine

## Abstract

**Background:**

Despite a growing body of scientific literature exploring the nature of meditation there is limited information on the characteristics of individuals who use it. This is particularly true of comparative studies examining prevalence and predictors of use of various forms of meditation.

**Methods:**

A secondary analysis was conducted using data from the 2012 National Health Interview Survey (*n* = 34,525). Three popular forms of meditation were compared—mantra, mindfulness, and spiritual—to determine lifetime and 12-month use related to key sociodemographic, health behavior, health status, and healthcare access variables.

**Results:**

The 12-month prevalence for meditation practice was 3.1% for spiritual meditation, 1.9% for mindfulness meditation, and 1.6% for mantra meditation. This represents approximately 7.0, 4.3, and 3.6 million adults respectively. A comparison across the three meditation practices found many similarities in user characteristics, suggesting interest in meditation may be more related to the type of person meditating than to the type of practice selected. Across meditation styles use was more prevalent among respondents who were female, non-Hispanic White, college educated, physically active; who used other complementary health practices; and who reported depression. Higher utilization of conventional healthcare services was one of the strongest predictors of use of all three styles. In addition to similarities, important distinctions were observed. For example, spiritual meditation practice was more prevalent among former drinkers. This may reflect use of spiritual meditation practices in support of alcohol treatment and sobriety. Reasons for use of meditation were examined using the sample of respondents who practiced mindfulness meditation. Wellness and prevention (74%) was a more common reason than use to treat a specific health condition (30%). Common reasons for use included stress management (92%) and emotional well-being (91%), and to support other health behaviors. Meditation was viewed positively because it was self-care oriented (81%) and focused on the whole person (79%).

**Conclusion:**

Meditation appears to provide an accessible, self-care resource that has potential value for mental health, behavioral self-regulation, and integrative medical care. Considering consumer preference for distinct types of meditation practices, understanding the underlying mechanisms, benefits, and applications of practice variations is important.

## Background

Meditation has played an important role in self-regulation and contemplative practice for millennia. In the United States interest in meditation and Eastern thought began to emerge as a more prominent aspect of American culture following the end of World War II [1, 2]. Searching the term “meditation” in the PubMed database today produces more than 4000 citations. Although there have been secular trends in research, beginning with Zen in the 1950s, Transcendental Meditation in the 1960s, and Mindfulness meditation in the 1970s, a large percentage of all meditation studies have been published in the last ten years, including the bulk of studies published using fMRI technology [3]. In addition to interest within the research community, national surveys consistently show meditation to rank as one of the most commonly used complementary health practices (CHP) among adults in the United States. Results from the National Health Interview Survey Adult Alternative Medicine supplement noted that meditation use in the previous 12 months was reported to be 7.6% of adults in 2002, and 9.7% in 2007. In 2012 reported adult use was 8.0% for analysis using the term “meditation” or 4.1% using a revised, more specific definition of meditation adopted for the 2012 NHIS. Meditation was consistently one of the top five complementary health practices across these three time periods [4–7]. Growing media coverage of meditation in English language print sources similarly reflects significant consumer interest [8].

Scientific research has contributed to an increased understanding of the mechanisms, effects, and applications of meditation. Meditation has been shown to be associated with alterations in brain structure [9–12], better mental health [13–17], improved attention [18, 19], greater emotional self-regulation [20–22], slower cellular aging [23–25], better academic performance [26, 27], and other outcomes. Growing evidence-based support for meditation has undoubtedly contributed to increased use of meditation in secular settings and applications. Meditation practices are now being integrated into psychotherapy for mental health [28–30], school-based programs to facilitate attention and socio-emotional development [31–33], corporate settings [34, 35], prisons [36], the military [37], drug and alcohol treatment programs [38, 39], and in hospitals for disease management and self-care [40–42]. Despite apparent benefits, however, a review of four hundred meditation clinical trials conducted between 1956 and 2005 found the methodology of many trials to be poor but improving, and noted the need for continued rigor in design and execution [43]. A review of meditation neuroscience research similarly noted that many of the studies had low methodological quality, used small samples, and that the majority of findings had not been replicated [44].

Despite persistent consumer interest, and an evolving body of literature examining the mechanisms and effects of meditation, there are very few studies characterizing the prevalence and patterns of use of meditation in the general US adult population. Certainly, one of the challenges in studying meditation, including the examination of consumer use, is that meditation is difficult to define. The absence of a clear operational definition is in part a function of the diversity of traditions, expressed in a profuse array of practices and beliefs, with fundamentally different ontological perspectives, embedded in unique cultural and historical contexts [45–48]. Despite these challenges, efforts are ongoing to more accurately define and operationalize meditation and delineate important similarities and differences between major styles of practice [21, 49–58].

In recognition of the diversity of traditions and practices, research staff at the National Institutes of Health (NIH) National Center for Complementary and Integrative Health (NCCIH) revised the National Health Interview Survey (NHIS) questions on meditation for the 2012 NHIS Adult Alternative Medicine supplement survey. The 2002 and 2007 surveys asked if respondents had ever used/used in the past 12 months various relaxation techniques, listing “meditation” as one of the options. To provide greater insight into patterns of meditation use, it was decided that the 2012 survey would collect data on three specific forms of meditation—mantra meditation, mindfulness meditation, and spiritual meditation. The supplement survey question revision process, including expert input and qualitative testing, has been documented [59].

The three types of meditation incorporated into the 2012 NHIS have Eastern and Western historical roots and represent popular contemporary forms of practice. These meditation practices may be generally characterized as follows:

Mantra meditation employs the use of a mentally repeated word or phrase, with the objective of maintaining attention on that specific object. As such, mantra meditation may be considered to be more of a focused attention style of practice [21]. The term *mantra* comes from Sanskrit and means instrument of thought, and sacred text [60]. The phrase “Om Mani Padme Hum” is a classic Tibetan Buddhist mantra. Use of mantra is found in many Eastern traditions, including Tibetan and Pure Land Buddhism, Sikhism, and Jainism. Transcendental meditation is a popular mantra style that employs traditional Sanskrit words. The Relaxation Response uses mental repetition of the English language word “one.” Some spiritual meditation practices may also employ silent repetition of a religious word or phrase.

Mindfulness meditation involves ongoing, non-reactive awareness or monitoring of the present moment, of one’s phenomenological experience. As such, mindfulness meditation has been described as more of an open monitoring style of practice [21]. The name of this meditation is derived from the Pali/Sanskrit terms *sati/smriti*, which mean, “to remember, bear in mind, be mindful of” [60]. Mindfulness is found in many Eastern traditions including Buddhist practices in China, Japan and Southeast Asia, as well as Taoist traditions. Mindfulness (Vipassana) and Zen are popular forms of mindfulness-oriented practices. Increasingly popular mindfulness-based therapies integrate mindfulness meditation and mindful awareness practices into psychotherapeutic applications.

Spiritual meditation focuses on developing a deeper understanding of spiritual/religious meaning and connection with a higher power. Spiritual meditation can be performed according to the practices of one of the major religious traditions or within a spiritual tradition. Examples include Christian contemplative prayer, Sufi dhikr, and Jewish kabbalistic practices. The techniques used in spiritual meditation may be the same as in other types of meditation, such as being attentive to a meditative word or phrase like “maranatha,” but the focus is on spiritual insight or connection. As some of these practices emphasize reflection on religious teachings they may be more similar to Eastern contemplative practices versus more traditional Eastern meditation practices, such as similarity with the Theravadan Buddhist *marananussati bhavana* practice, or reflection on one’s mortality, described in the *Visuddhimagga* commentary. Also, some individuals may consider prayer to be a form of meditation. Although the 2002 and 2007 NHIS asked about prayer practices, the 2012 NHIS did not ask specifically about religious identity or use of other religious/spiritual practices, such as prayer.

An excellent overview of meditation use, based on the revised items used in the 2012 NHIS survey, provided important information on the characteristics of individuals who practiced any of the three newly specified forms of meditation (12-month use, 4.1%) [7]. One challenge as noted by the authors, however, was the significant overlap among the three practices found in the survey data. They noted that 32% of respondents reported practicing two methods, and 18% reported use of all three methods, some 50% mixing practices. This overlap may be obscuring insight into important differences between these three types of meditation. To date, there is very little published research of any sort directly comparing different types of meditation. Among them, a number of studies have found individual differences related to preference for type of meditation as well as differential effects from various forms of meditation [61, 62]. The other significant gap is published studies using national data comparing prevalence and characteristics of use of common styles of meditation. For this reason an analysis was conducted using the 2012 NHIS survey, with particular focus on comparing individuals who exclusively practiced one of the three forms of meditation newly specified in the 2012 data. It was hypothesized that meditators, as compared with non-meditators, would have demographics similar to users of other complementary health practices; that spiritual meditation would differ from the other two practices on a variety of key characteristics; and that reasons for use of mindfulness meditation would emphasize wellness versus treatment.

## Methods

### Population of study

Analyses in this article were performed using data from the Adult Alternative Medicine supplement, the Sample Adult Core, and the Family Core components of the 2012 NHIS. The NHIS is conducted annually by the National Center for Health Statistics (NCHS). The 2012 NHIS consisted of core questions, questions on healthcare access and utilization, and additional supplementary questions on a range of subjects, such as use of complementary health practices.

The 2012 NHIS interview sample included 42,366 households, consisting of 108,131 persons in 43,345 families. The household response rate was 77.6%, with 34,525 adults completing interviews, resulting in a sample adult response rate of 61.2%. All sample adults were also interviewed about the use of more than twenty different complementary health practices. The 2012 NHIS was approved by the National Center for Health Statistics Research Ethics Review Board. Verbal consents were obtained from all survey respondents. More information on the NHIS can be found at: www.cdc.gov/nchs/nhis.htm [63].

### Dependent variables

In the 2012 NHIS Adult Alternative Medicine supplement, respondents were asked about three types of meditation use—mantra, mindfulness and spiritual meditation. Survey participants could respond that they used one, two, or all three meditation practices. To inquire about meditation practice respondents were given a prompt for each type of meditation, which included the name, such as mantra, and examples of types that would be included in that domain, such as Transcendental Meditation.

### Independent variables

A number of items were included as predictor variables based on known and hypothesized associations with the use of complementary health practices [4–6, 64–66]. These items included eight sociodemographic characteristics, five health behaviors and four complementary health practices, seven health status variables, and three related to healthcare access. These variables have been described in more detail in previously published work [67].

In addition, in the event that adults used more than three complementary health practices (3.2% of respondents in the Sample Adult survey sample and 10.9% of the Alternative Medicine supplement sample), they were asked to select the three that they felt were the most important approaches used for their health, and then asked to report reasons for use of each of those three approaches. Reasons were not mutually exclusive and respondents could report both wellness and treatment reasons. Several of these reason-for-use items were included as variables for analysis of the subsample of respondents who selected mindfulness meditation as one of their top three therapies. Predictor variable responses of “refused,” “not ascertained,” and “don’t know” were treated as missing data and excluded from analysis.

### Statistical analyses

To compare meditators and non-meditators on key sociodemographic and health variables all three meditation styles were combined into a single meditation variable. This was done by selecting individuals from the Adult Alternative Medicine supplement sample who had reported “yes” for use of any of the three meditation choices in the previous 12 months (mantra, mindfulness, or spiritual). Use within the 12-month timeframe was selected for all analyses as it captured both new and longer-term practice. Meditators and non-meditators were compared on key predictor variables in the four categories of interest: demographics, health behaviors (including use of other complementary health practices), health status, and healthcare access (Table 1). The unadjusted associations were tested by chi-square statistics.

**Table 4:** Reasons for practicing mindfulness meditation, exclusive use during the previous 12 months

	Percent^a^	SE	95% CI
General Use			
For general wellness and disease prevention	73.3	5.2	63.0, 83.7
To treat specific health problems	30.2	5.0	20.3, 40.2
Wellness Use			
Reduce stress levels or relaxation	91.8	4.1	83.7, 99.9
Feel better emotionally	90.5	3.7	83.2, 97.9
Sense of control over one’s health	65.1	6.1	52.9, 77.3
Improve memory or concentration	56.8	7.0	42.9, 70.7
Better sleep	55.7	7.0	42.0, 69.5
Improve energy	43.4	6.0	31.3, 55.4
Improve immune function	27.7	6.0	15.7, 39.8
Health Behavior Use			
Eat healthier	31.9	6.7	18.5, 45.3
Cut back or stop smoking cigarettes	26.8	8.1	10.0, 43.3
Exercise more regularly	26.3	5.5	15.3, 37.4
Eat more organic foods	9.7	3.5	2.8, 16.6
Cut back or stop drinking alcohol	8.2	3.3	1.6, 14.7
Health Problem Use			
Can be practiced/done on my own	81.4	5.5	70.4, 92.5
Whole person focus—mind, body, and spirit	79.3	5.1	69.2, 89.3
It is natural	72.5	5.9	60.7, 84.2
Would be helpful combined with medical treatment	70.2	7.5	54.6, 85.7
Treats the cause and not just the symptoms	60.6	6.4	47.8, 73.4
Medications cause side effects	43.9	7.4	28.3, 59.4
Medical treatments not effective for my problem	26.4	11.6	2.3, 50.5
Recommended by a medical doctor	13.0	3.9	5.2, 20.7

^a^Percent is age adjusted

Prevalence patterns in relation to the sociodemographic and health variables were then examined in order to help characterize and differentiate the three methods (Table 2). For this analysis three subsamples were created. Each subsample was comprised of individuals who had exclusively practiced mantra, mindfulness, or spiritual meditation within the past 12 months.

**Table 1:** Comparison of meditators and non-meditators by select user characteristics, use during the previous 12 months (age adjusted and standardized)^a^

	Meditators^b^ (4.10%)	Non-Meditators (95.90%)	*p*-value
	Age-adjusted Percent (SE)	Age-adjusted Percent (SE)	
Demographics			
Age^c^			
18–24	10.15 (1.26)	13.03 (0.33)	0.040
25–44	37.08 (1.81)	34.40 (0.39)	0.139
45–64	42.23 (1.84)	34.54 (0.37)	<0.001
65 or above	10.55 (1.18)	18.04 (0.29)	<0.001
Gender			
Male	38.55 (1.88)	48.65 (0.37)	<0.001
Female	61.45 (1.88)	51.35 (0.37)	
Race^d^			
White	86.30 (1.11)	80.96 (0.33)	<0.001
Black	8.05 (0.87)	12.37 (0.28)	<0.001
Asian	3.87 (0.60)	5.47 (0.18)	0.040
AIAN/multiple race	1.78 (0.45)	1.21 (0.10)	0.156
Ethnicity			
Hispanic	8.19 (0.90)	15.40 (0.36)	<0.001
Non-Hispanic	91.81 (0.90)	84.60 (0.36)	
Education			
Less than 12 years	4.53 (0.84)	14.45 (0.29)	<0.001
High school graduate	10.68 (1.06)	26.79 (0.37)	<0.001
Some college	23.60 (1.43)	20.48 (0.33)	0.192
College graduate	61.19 (1.55)	38.27 (0.38)	<0.001
Marital status			
Never married	27.18 (1.24)	22.33 (0.25)	0.012
Married	46.56 (1.60)	53.27 (0.34)	0.004
Cohabitating	8.45 (0.94)	7.42 (0.19)	0.161
Divorced or separated	14.24 (1.07)	11.11 (0.02)	<0.001
Widowed	3.57 (0.60)	5.86 (0.14)	<0.001
Family income^e^			
$0–34,999	30.22 (1.54)	33.33 (0.43)	0.007
$35,000–54,999	30.75 (1.77)	32.11 (0.37)	0.498
$75,000 or more	39.02 (1.91)	34.56 (0.46)	0.004
Region			
Northeast	17.70 (1.52)	18.23 (0.37)	0.386
Midwest	23.58 (1.70)	22.66 (0.41)	0.365
South	26.15 (2.03)	36.83 (0.48)	<0.001
West	32.56 (1.86)	22.28 (0.44)	<0.001
Health Behaviors			
Physical activity			
Never	23.36 (1.58)	44.00 (0.46)	<0.001
Some/per week	52.66 (1.99)	40.97 (0.44)	<0.001
Regular/per day	23.98 (1.64)	15.03 (0.33)	<0.001
Alcohol consumption			
Lifetime abstainer	9.91 (1.22)	21.46 (0.35)	<0.001
Former drinker	13.52 (1.18)	14.44 (0.28)	0.335
Current infrequent	51.11 (1.79)	43.95 (0.37)	<0.001
Current moderate/heavy	25.47 (1.59)	20.16 (0.34)	<0.001
Cigarette smoking			
Never smoker	53.89 (1.13)	60.09 (0.37)	0.001
Former smoker	27.79 (1.43)	21.81 (0.29)	<0.001
Current smoker	18.33 (1.13)	18.10 (0.30)	0.331
Cholesterol check, 12 months	66.11 (1.52)	61.93 (0.33)	0.040
Flu shot, 12 months	38.56 (1.63)	36.85 (0.34)	0.717
*CAM Use, 12 Months*			
Chiropractor	17.39 (1.29)	8.07 (0.21)	<0.001
Acupuncture	7.10 (0.77)	1.26 (0.07)	<0.001
Yoga	45.88 (1.82)	7.73 (0.21)	<0.001
Vegetarian diet	11.42 (1.10)	1.54 (0.09)	<0.001
Health Status			
Health status			
Poor/fair	11.46 (1.11)	12.80 (0.24)	0.264
Good/very good/excellent	88.54 (1.11)	87.20 (0.24)	
Body weight status			
Underweight/healthy	41.18 (1.81)	34.96 (0.35)	<0.001
Overweight	32.50 (1.67)	33.65 (0.36)	0.434
Obese	26.33 (1.57)	31.39 (0.36)	0.003
Functional limitations	45.35 (1.59)	33.76 (0.35)	<0.001
Back pain	38.53 (1.74)	27.44 (0.33)	<0.001
Depression, 12 months	21.80 (1.42)	9.46 (0.21)	<0.001
Emergency room visits, 12 months			
0 visit	77.24 (1.36)	80.72 (0.32)	0.006
1 visit	13.67 (1.12)	12.14 (0.24)	0.065
2–3 visits	7.35 (0.83)	6.24 (0.19)	0.409
4 or More Visits	1.75 (0.48)	0.90 (0.07)	0.013
Hospitalized, 12 Months	9.99 (1.10)	9.10 (0.22)	0.606
Healthcare Access			
Usual place of care	85.53 (1.09)	83.64 (0.30)	0.155
Health insurance			
Uninsured	14.73 (1.10)	17.05 (0.31)	0.384
Public	33.45 (1.18)	33.86 (0.37)	0.014
Private	51.82 (1.62)	49.09 (0.41)	0.001
Office visits, 12 months			
0 visit	10.64 (0.98)	20.28 (0.32)	<0.001
1–3 visits	37.71 (1.64)	44.02 (0.38)	0.001
4–9 visits	25.76 (1.55)	22.85 (0.31)	0.053
10 or more visits	25.89 (1.56)	12.85 (0.24)	<0.001

^a^Age adjusted by using the projected 2000 US population as the standard population with the following age groups: 18–24, 25–44, 45–64, ≥65

^b^Meditators include any use of mantra, mindfulness and spiritual meditation, past 12 months

^c^Age categories were not standardized to the 2000 population

^d^Race category “not releasable” were not displayed due to small sample size

^e^Estimates were generated using the first imputed income file

*AIAN* American Indian and Alaska Native

For the multivariable logistic regression analyses, the sample of individuals who reported exclusive use of mantra, mindfulness, or spiritual meditation in the previous 12 months was used (Table 3). Given the large sample size and the somewhat exploratory nature of this analysis, all items included in Table 1 were entered into the regression analysis, except for use of yoga, as several Alternative Medicine survey questions comingled meditation and yoga resulting in high multicollinearity.

**Table 2:** Prevalence of meditation use by select user characteristics, exclusive use of one practice during previous 12 months^1^

	*n* ^3^	Mantra	*n*	Mindfulness	*n*	Spiritual^2^
		Age-adjusted Percent (SE)		Age-adjusted Percent (SE)		Age-adjusted Percent (SE)
Exclusive use	122	0.33 (0.04)	151	0.44 (0.49)	498	1.48 (0.09)
Estimated use (1000s)	740		998		3359	
Demographics						
Age^4^						
18–24	9	†	22	0.52 (0.14)a	40	1.02 (0.23)a
25–44	45	0.37 (0.06)a	67	0.50 (0.08)a	161	1.44 (0.15)a
45–64	54	0.37(0.06)a	53	0.51 (0.10)a	218	1.87 (0.16)b
65 or above	14	0.21 (0.07)a‡	9	0.13 (0.05)b‡	79	1.14 (0.18)a
Gender						
Male	41	0.25 (0.05)a	54	0.36 (0.07)a	152	1.04 (0.12)a
Female	81	0.40 (0.06)b	97	0.52 (0.08)a	346	1.89 (0.13)b
Race^5^						
White	106	0.36 (0.05)a	125	0.49 (0.06)a	394	1.56 (0.11)a
Black	6	†	10	0.16 (0.06)b‡	71	1.27 (0.19)a,b
Asian	9	0.24 (0.09)a‡	9	0.31 (0.10)a,b	22	0.86 (0.19)b
AIAN/multiple race	1	†	6	0.56 (0.24)a,b‡	10	1.43 (0.49)a,b‡
Ethnicity						
Hispanic	11	0.18 (0.06)a	14	0.20 (0.07)a	51	0.95 (0.19)a
Non-Hispanic	111	0.35 (0.05)b	137	0.49 (0.06)b	447	1.59 (0.10)b
Education						
Less than 12 years	7	†	4	†	19	0.40 (0.12)a
High school graduate	14	0.10 (0.03)a	19	0.19 (0.06)a	72	0.85 (0.13)b
Some college	25	0.35 (0.09)b	27	0.32 (0.08)a	109	1.68 (0.20)c
College graduate	76	0.48 (0.07)b	101	0.73 (0.10)b	297	2.23 (0.17)d
Marital status						
Never married	34	0.61 (0.15)a	50	0.41 (0.87)a	132	1.87 (0.24)a
Married	57	0.29 (0.50)b	54	0.35 (0.06)a	201	1.30 (0.13)b
Cohabitating	7	0.20 (0.07)b‡	13	0.49 (0.17)a	22	1.09 (0.27)b
Divorced or separated	18	0.22 (0.06)b	29	0.45 (0.09)a	113	1.96 (0.28)a
Widowed	6	†	5	†	29	1.27 (0.55)a,b
Family income^6^						
$0–34,999	42	0.33 (0.06)a	50	0.34 (0.08)a	190	1.37 (0.12)a
$35,000–54,999	40	0.31 (0.06)a	47	0.38 (0.07)a	154	1.52 (0.16)a
$75,000 or more	40	0.37 (0.08)a	54	0.55 (0.11)a	154	1.61 (0.18)a
Region						
Northeast	28	0.43 (0.10)a,b	25	0.41 (0.10)a	94	1.53 (0.19)a,b
Midwest	20	0.30 (0.09)a,b	28	0.40 (0.09)a	113	1.77 (0.24)a
South	26	0.22 (0.05)a	25	0.17 (0.06)b‡	139	1.19 (0.14)b
West	48	0.46 (0.10)b	73	0.93 (0.16)c	152	1.63 (0.17)a
Health Behaviors						
Physical activity						
Never	37	0.21 (0.04)a	33	0.24 (0.07)a	135	0.99 (0.11)a
Some/per week	60	0.41 (0.07)b	89	0.60 (0.08)b	250	1.84 (0.16)b
Regular/per day	23	0.42 (0.10)b	25	0.53 (0.14)a,b	105	1.96 (0.24)b
Alcohol consumption						
Lifetime abstainer	6	0.04 (0.02)a‡	8	0.10 (0.04)a‡	75	1.09 (0.18)a
Former drinker	12	0.16 (0.06)b‡	11	†	86	2.01 (0.28)b
Current infrequent	74	0.48 (0.07)c	75	0.52 (0.07)b	232	1.54 (0.14)a,b
Current mod/heavy	30	0.37 (0.85)c	56	0.74 (0.13)b	102	1.59 (0.22)a,b
Cigarette smoking						
Never smoker	71	0.34 (0.05)a	80	0.42 (0.06)a	260	1.33 (0.11)a
Former smoker	26	0.29 (0.06)a	36	0.43 (0.10)a	138	2.11 (0.28)b
Current smoker	25	0.33 (0.08)a	34	0.40 (0.10)a	100	1.34 (0.18)a
Cholesterol check						
Yes	42	0.39 (0.06)a	59	0.46 (0.07)a	155	1.49 (0.12)a
No	80	0.28 (0.06)a	92	0.42 (0.08)a	339	1.21 (0.13)a
Flu shot						
Yes	76	0.32 (0.06)a	101	0.46 (0.09)a	303	1.65 (0.16)a
No	46	0.34 (0.05)a	50	0.43 (0.05)a	195	1.39 (0.10)a
*CAM Use, 12 Months*						
Chiropractor						
Yes	97	0.72 (0.17)a	133	0.43 (0.12)a	406	3.09 (0.43)a
No	25	0.29 (0.04)b	18	0.44 (0.05)a	91	1.32 (0.09)b
Acupuncture						
Yes	107	3.11 (1.12)a	142	2.29 (0.72)a	476	3.41 (0.93)a
No	15	0.29 (0.04)b	9	0.42 (0.72)b	22	1.45 (0.09)b
Yoga						
Yes	64	1.94 (0.24)a	68	2.79 (0.39)a	350	4.82 (1.16)a
No	58	0.18 (0.03)b	82	0.20 (0.03)b	148	1.40 (0.09)b
Vegetarian diet						
Yes	109	1.43 (0.51)a	132	2.72 (0.78)a	458	6.11 (1.13)a
No	12	0.30 (0.04)b	18	0.39 (0.05)b	40	1.40 (0.09)b
Health Status						
Health status						
Poor/fair	16	0.32 (0.12)a‡	22	0.46 (0.14)a	77	1.52 (0.24)a
Good/very/excellent	106	0.33 (0.04)a	129	0.44 (0.05)a	420	1.48 (0.10)a
Body weight status						
Underweight/healthy	47	0.34 (0.07)a	71	0.67 (0.11)a	177	1.50 (0.14)a
Overweight	34	0.25 (0.05)a	47	0.43 (0.08)a,b	158	1.48 (0.15)a
Obese	39	0.37 (0.09)a	29	0.23 (0.07)b	158	1.46 (0.14)a
Functional limits						
Not limited	56	0.27 (0.04)a	63	0.40 (0.06)a	239	1.19 (0.09)b
Limited	66	0.46 (0.08)a	88	0.58 (0.12)a	259	2.23 (0.22)a
Back pain						
Yes	70	0.47 (0.08)a	94	0.50 (0.09)a	282	2.18 (0.18)a
No	52	0.26 (0.04)b	57	0.42 (0.06)a	216	1.23 (0.10)b
Depression, 12 months						
Yes	92	0.69 (0.15)a	117	0.87 (0.20)a	379	3.10 (0.35)a
No	30	0.28 (0.04)b	34	0.39 (0.05)b	118	1.30 (0.09)b
ER visits, 12 months						
0 visit	89	0.29 (0.04)a	119	0.43 (0.05)a	377	1.44 (0.10)a
1 visit	21	0.57 (0.14)a	23	0.59 (0.18)a	68	1.54 (0.24)a
2–3 visits	10	0.40 (0.13)a	7	0.19 (0.08)b‡	41	1.84 (0.36)a
4 or More Visits	2	†	2	†	12	2.69 (0.97)a
Hospitalized, 12 Months						
Yes	106	0.46 (0.13)a	141	0.26 (0.11)a‡	448	1.43 (0.26)a
No	16	0.31 (0.04)a	10	0.46 (0.05)a	49	1.49 (0.10)a
Healthcare Access						
Usual place of care						
Yes	16	0.35 (0.04)a	28	0.45 (0.54)a	64	1.55 (0.10)a
No	106	0.21 (0.08)a	123	0.35 (0.09)a	434	1.14 (0.20)a
Health insurance						
Uninsured	17	0.17 (0.05)a	21	0.29 (0.08)a	73	1.41 (0.29)a
Public	50	0.53 (0.11)b	49	0.49 (0.10)a	187	1.41 (0.16)a
Private	55	0.29 (0.05)a,b	81	0.47 (0.07)a	236	1.52 (0.15)a
Office visits, 12 months						
0 visit	15	0.12 (0.04)a‡	20	0.17 (0.04)a	49	0.73 (0.14)a
1–3 visits	39	0.23 (0.05)a,b	59	0.45 (0.07)b	170	1.28 (0.13)b
4–9 visits	31	0.44 (0.13)b,c	33	0.39 (0.11)a,b	155	2.02 (0.21)c
10 or more visits	37	0.81 (0.16)c	39	1.01 (0.22)c	124	2.56 (0.29)c

^1^Age adjusted by using the projected 2000 US population as the standard population with the following age groups: 18–24, 25–44, 45–64, ≥65

^2^Spiritual meditation prevalence is significantly higher than mantra and mindfulness meditation across most variables, except for age 18–24, AIAN/multiple race, and acupuncture use

^3^n reflects the count at each category from the sample data

^4^Age categories were not standardized to the 2000 population

^5^Race category “not releasable” were not displayed due to small sample size

^6^Estimates were generated using the first imputed income file

†Estimates with a relative S.E. >50% are not shown

‡Estimates with a relative S.E. >30% but ≤50% unstable because of the small sample size

a,b,c,d Within-group comparisons were performed across all nominal variables within each meditation type, α = 0.05 was used for all comparisons. Different superscript letters represent a statistically significant difference between the within-group categories compared, and same superscript letters indicate no statistical difference.

*AIAN* American Indian and Alaska Native

*ER* Emergency Room

Finally, an analysis was conducted to provide information on respondents’ reasons for use of meditation in terms of wellness/self-care and treatment of health conditions (Table 4). Mindfulness meditation, 12-month exclusive use, was selected for this analysis as it is growing in use among consumers, it is currently the most actively researched meditation method, and variants of the method have been adopted into diverse secular settings. Alcohol consumption and smoking behaviors were only asked of adults who reported that they were current drinkers or smokers in the NHIS Core questionnaire.

**Table 3:** Multivariable logistic regression predicting meditation practices, exclusive use of one meditation style during the previous 12 months

	Mantra	Mindfulness	Spiritual
	OR (95% CI)	OR (95% CI)	OR (95% CI)
Demographics			
Age			
18–24	Reference		
25–44	1.13 (0.38, 3.39)	0.70 (0.34, 1.42)	1.19 (0.69, 2.08)
45–64	1.47 (0.47, 4.59)	0.76 (0.36, 1.62)	1.31 (0.74, 2.32)
65 or above	0.76 (0.18, 3.16)	0.19 (0.05, 0.71)*	1.10 (0.53, 2.26)
Gender			
Male	Reference		
Female	1.11 (0.64, 1.92)	1.17 (0.63, 2.16)	1.54 (1.15, 2.06)**
Race^1^			
White	Reference		
Black	0.37 (0.12, 1.17)	0.51 (0.22, 1.22)	0.91 (0.64, 1.30)
Asian	0.85 (0.36, 2.01)	0.51 (0.23, 1.11)	0.62 (0.38, 1.01)
AIAN/multiple race	0.37 (0.05, 3.04)	2.01 (0.75, 5.33)	1.09 (0.52, 2.30)
Ethnicity			
Hispanic	Reference		
Non-Hispanic	0.66 (0.28, 1.52)	0.62 (0.30, 1.25)	0.95 (0.62, 1.46)
Education			
Less than 12 years	Reference		
High school	0.52 (0.16, 1.73)	1.91 (0.47, 7.78)	2.08 (1.07, 4.05)*
Some college	1.49 (0.51, 4.35)	2.98 (0.75, 11.82)	3.85 (2.02, 7.32)***
College graduate	2.04 (0.71, 5.88)	6.61 (1.70, 25.74)**	5.52 (2.91, 10.47)***
Marital status			
Never married	Reference		
Married	0.71 (0.36, 1.39)	0.86 (0.49, 1.52)	0.68 (0.48, 0.94)*
Cohabitating	0.54 (0.22, 1.34)	0.65 (0.27, 1.53)	0.71 (0.40, 1.27)
Divorced/separated	0.40 (0.18, 0.86)*	0.92 (0.49, 1.74)	0.93 (0.66, 1.32)
Widowed	0.42 (0.13, 1.36)	1.38 (0.44, 4.40)	0.44 (0.24, 0.80)**
Family income			
$0–$34,999	Reference		
$35,000–54,999	0.71 (0.42, 1.22)	1.15 (0.61, 2.18)	1.08 (0.80, 1.44)
$75,000 or more	0.55 (0.24, 1.24)	1.19 (0.55, 2.56)	1.12 (0.78, 1.59)
Region			
Northeast	Reference		
Midwest	0.58 (0.27, 1.26)	0.86 (0.46, 1.60)	1.06 (0.72, 1.54)
South	0.61 (0.32, 1.17)	0.48 (0.21, 1.07)	0.77 (0.54, 1.10)
West	0.99 (0.53, 1.87)	1.96 (1.09, 3.51)*	0.96 (0.67, 1.38)
Health Behaviors			
Physical activity			
Never	Reference		
Some/per week	1.37 (.83, 2.26)	1.54 (0.84, 2.80)	1.54 (1.13, 2.10)**
Regular/per day	1.60 (.87, 2.95)	1.41 (0.65, 3.02)	1.59 (1.10, 2.29)*
Alcohol consumption			
Lifetime abstainer	Reference		
Former drinker	4.52 (1.26, 16.28)*	1.61 (0.44, 5.92)	1.28 (0.79, 2.08)
Current infrequent	10.80 (3.73, 31.27)***	3.34 (1.32, 8.45)*	0.86 (0.56, 1.32)
Current mod/heavy	9.63 (3.02, 30.69)***	4.09 (1.54, 10.85)**	0.90 (0.55, 1.46)
Cigarette smoking			
Never smoker	Reference		
Former smoker	0.61 (0.37, 1.02)	1.03 (.62, 1.69)	1.48 (1.10, 2.00)*
Current smoker	0.83 (0.45, 1.50)	1.07 (0.59, 1.95)	1.34 (0.92, 1.94)
Cholesterol check	1.04 (0.63, 1.72)	0.84 (0.48, 1.47)	0.85 (0.66, 1.11)
Flu shot	0.66 (0.37, 1.18)	0.85 (0.52, 1.38)	1.01 (0.78, 1.29)
*CAM Use, 12 Months*			
Chiropractor	1.09 (0.57, 2.08)	0.40 (0.19, .82)*	1.36 (0.98, 1.90)
Acupuncture	5.17 (2.35, 11.40)***	1.62 (0.79, 3.30)	1.22 (0.65, 2.31)
Vegetarian diet	3.28 (1.45, 7.44)**	3.41 (1.54, 7.58)**	2.63 (1.67, 4.14)***
Health Status			
Health status			
Poor/fair	Reference		
Good/very/excellent	1.70 (0.76, 3.81)	0.70 (0.32, 1.51)	1.13 (0.74, 1.72)
Body weight status			
Underweight/healthy	Reference		
Overweight	0.97 (0.55, 1.72)	0.79 (0.48, 1.33)	1.10 (0.83, 1.44)
Obese	1.43 (0.72, 2.81)	0.35 (0.17, 0.75)**	1.04 (0.80, 1.35)
Functional limitations	1.17 (0.70, 1.98)	1.17 (0.75, 1.83)	1.53 (1.11, 2.11)*
Back pain	1.26 (0.81, 1.98)	0.83 (0.50, 1.38)	1.31 (1.02, 1.68)*
Depression, 12 months	1.73 (0.97, 3.07)	2.08 (1.09, 3.96)*	1.65 (1.22, 2.21)**
ER visits, 12 months			
0 visits	Reference		
1 visit	1.50 (0.78, 2.88)	1.48 (0.78, 2.81)	0.80 (0.55, 1.16)
2–3 visits	0.92 (0.42, 2.04)	0.29 (0.09, 1.00)	0.98 (0.61, 1.57)
4 or More Visits	0.12 (0.01, 1.02)	2.42 (0.53, 11.10)	1.61 (0.71, 3.65)
Hospitalized, 12 Months	1.08 (0.54, 2.15)	0.31 (0.11, 0.86)*	0.74 (0.49, 1.11)
Healthcare Access			
Usual place of care	1.35 (0.57, 3.22)	0.72 (0.35, 1.50)	0.91 (0.61, 1.35)
Health insurance			
Uninsured	Reference		
Public	1.55 (0.68, 3.57)	0.91 (0.41, 2.01)	0.66 (0.44, 0.97)*
Private	0.79 (0.33, 1.91)	0.70 (0.34, 1.45)	0.78 (0.52, 1.16)
Office visits, 12 months			
0 visits	Reference		
1–3 visits	1.19 (0.48, 2.97)	2.79 (1.44, 5.38)**	1.66 (1.04, 2.65)*
4–9 visits	2.05 (0.76, 5.50)	2.39 (.86, 6.63)	2.27 (1.32, 3.90)**
10 or more visits	3.44 (1.28, 9.22)*	8.62 (3.77, 19.73)***	2.59 (1.47, 4.55)**

^1^Race category “not releasable” were not displayed due to small sample size

^2^Family income used all 5 imputed income files from NHIS

*AIAN* American Indian and Alaska Native

*ER* Emergency Room

*p < 0.05, **p < 0.01, ***p < 0.001 (compared to reference category)

Population estimates in the analyses were age-adjusted and calculated using sampling weights to represent the non-institutionalized population of US adults aged 18 and over. Data weighting procedures are described in more detail elsewhere [68]. Statistical tests were performed using Stata 13 [69], a software package that accounts for the complex sample design of NHIS data. Descriptive statistics, Student’s *t*-tests, Pearson’s chi-squared tests, and multivariable logistic regressions were conducted for the statistical analyses. All statistical analyses used two-tailed tests with α set to 0.05.

## Results

### Comparing meditators and non-meditators on key characteristics

Meditators and non-meditators were compared on key sociodemographic and health variables (Table 1). The meditator sample was comprised of respondents who used any of the three meditation practices during the previous 12 months. Meditators (4.1%, representing 9 million adults) differed significantly from non-meditators (95.9%, representing 218 million adults) on key sociodemographic variables including age, gender, race, ethnicity, educational attainment, and region of the country, typically at the *p* < .001 level. Looking at statistically significant differences the meditator sample had more middle aged respondents (45–65 years of age, 42% vs 35%) and fewer seniors (65 or older, 11% vs 18%), as well as more respondents who were female (61% vs 51%), White (86% vs 81%), college graduates (61% vs 38%), living in the West (33% vs 22%) but not in the South (26% vs 37%), and with higher family income ($75,000 or more, 39% vs 35%). The two groups differed significantly on the health behaviors of physical activity, alcohol consumption, cigarette smoking, and cholesterol screening, but they did not differ in recent flu shot status. For example, only 23% of meditators reported engaging in no regular physical activity compared with 44% of non-meditators (*p* < .001). Only 10% of meditators reported lifetime abstinence from alcohol compared with 21% of non-meditators, and more meditators reported being former smokers (28% vs 22%). Meditators were also more likely to use all CHP methods listed, including provider-based methods such as acupuncture and chiropractic/osteopathic, and self-care methods including yoga and vegetarian diets. For example, 11% of meditators reported use of vegetarian diet in the previous 12 months versus 2% of non-meditators (*p* < .001).

For health status, meditators and non-meditators differed significantly in body mass index (BMI), functional limitations, emergency room visits, chronic back pain, and depression, but not in hospitalizations in the past year or self-reported health status. For BMI, meditators were less likely to report obesity (26% vs 31%, *p* < .003). In contrast to this, more meditators reported one or more functional limitations (45% vs 34%), chronic back pain (39% vs 27%), and depression (22% vs 9%), all at *p* < .001. The two groups also differed significantly on healthcare access in terms of number of visits to conventional healthcare providers in the previous year, and type of health insurance, but no difference in terms of access to a usual place of care. More meditators reported 10 or more visits to a conventional healthcare provider in the last year compared to non-meditators (26% vs 13%, *p* < .001).

### Comparing across the three meditation practices on key characteristics

Survey respondents provided information on lifetime use and 12-month use of mantra, mindfulness and spiritual meditation. Respondents could report using one, two or all three of these methods during these two time periods. For overall lifetime use, 5.3% reported practicing one or more of the meditation methods (approximately 11.9 million adults). Looking at the specific meditations, the largest number of respondents reported practicing spiritual meditation (3.7%), followed by mantra (2.6%), and mindfulness (2.5%). This represents 8.4, 5.9, and 5.7 million adults respectively (data not in tables). For overall use during the previous 12 months, the largest percentage of respondents reported using spiritual meditation (3.1%), followed by mindfulness (1.9%), and mantra (1.6%). These percentages represent approximately 7 million adults for spiritual meditation, 4.3 million for mindfulness, and 3.6 million for mantra. Within each of the three meditation practices, there was also a subsample of individuals who reported exclusive use of just one of the three practices. The sample sizes for 12-month exclusive use were: spiritual (1.5%), mindfulness (0.4%), and mantra (0.3%) representing approximately 3.4 million, 953,000 and 748,000 adults respectively.

Table 2 presents meditation prevalence information on the sample of individuals who reported exclusive use of mantra, mindfulness, or spiritual meditation during the 12 months prior to the survey interview. There was a great deal of similarity in the sociodemographic and health variable characteristics across the three styles of meditation. Examining the sociodemographic variables, for example, there was a higher prevalence of all three meditations among respondents who were female, Non-Hispanic, and who had higher educational attainment. In terms of region, mantra and mindfulness were most prevalent in the West, while spiritual meditation was highest in the Midwest and West. Prevalence was lowest in the South for all three groups.

Similarities across the three groups were also found for key health behaviors. For example, meditation was more common among respondents who were physically active, and who used other complementary health practices—acupuncture, yoga and vegetarian diet, with the exception of no significant difference in use of chiropractic/osteopathic for mindfulness meditators. Mindfulness and mantra meditation were more prevalent among those who consumed alcohol infrequently or moderately. Spiritual meditation was more prevalent for those reporting being a former drinker. Spiritual meditation was also more common among former smokers compared with current or never smoked cigarettes. In terms of health status, prevalence for all three styles of meditation was significantly higher for those reporting depression compared with non-depressed. Mantra and spiritual meditation, but not mindfulness, were more common among those reporting back pain. The only healthcare access variable with consistently significant differences for all three meditations was the number of office visits to a conventional provider in the 12 months prior to the survey. Meditation use was more prevalent among respondents who utilized more care.

### Multivariable logistic regression predicting the three meditation practices

A multivariable logistic regression analysis was performed with the same sample of individuals who reported exclusive use of manta, or mindfulness, or spiritual meditation in the previous 12 months (Table 3).

Mantra meditation included a number of significant predictors. For demographic variables, divorced individuals were less likely to practice mantra meditation compared with respondents who were not married. For health behaviors, former, infrequent or moderate drinkers were more likely to practice compared with lifetime abstainers (e.g. former drinker, OR = 4.5, 95% CI 1.3–16.3, *p* < .05). Complementary health practice variables strongly associated with mantra meditation practice included acupuncture (OR = 5.2, 95% CI 2.4–11.4, *p* < .001) and having used a vegetarian diet (OR = 3.3, 95% CI 1.5–7.4, *p* < .01). In terms of health status variables, neither back pain nor depression is a significant predictor. Four or more emergency room visits were marginally significant, with lower likelihood compared to no visits (*p* = .052). There was only one healthcare access variable significantly associated with mantra meditation and that was 10 or more conventional medicine office visits (OR = 3.4, 95% CI 1.3–9.2, *p* < .05).

Mindfulness meditation included several significant predictors. For demographic variables, participants 65 or older were significantly less likely to practice mindfulness compared to those aged 18–24. College graduates were more likely to practice mindfulness than individuals without a high school diploma (OR = 6.6, 95% CI 1.7–25.7, *p* < .01). Living in the West was also predictive of use (OR = 2.0, 95% CI 1.1–3.5, *p* < .05) compared with living in the Northeast. The only significant association for health behavior was infrequent and moderate alcohol consumption compared with lifetime abstinence. Complementary health practice items included a lower likelihood of chiropractic/osteopathic care, no association with acupuncture, and a higher likelihood of vegetarian diet (OR = 3.4, 95% CI 1.5–7.6, *p* < .01). In terms of health status, obese participants were less likely to practice mindfulness meditation (OR = 0.4, 95% CI 0.2–0.8, *p* < .01) compared with healthy/underweight. Back pain was not significant, but depression was (OR = 2.1, 95% CI 1.1–4.0, *p* < .05). Hospitalization in the previous year was less likely, and 2–3 emergency room visits was marginally significant (*p* = 0.051) compared with no visits. Similar to mantra, visits to a conventional medical provider was highly significant (10+ visits, OR = 8.6, 95% CI 3.8–19.7, *p* < .001).

Spiritual meditation had several significant predictors. For demographic variables, females, and those having a high school degree or greater were more likely to practice, while being married reduced the likelihood. The strongest association was with being a college graduate (OR = 5.5, 95% CI 2.9–10.5, *p* < .001). For health behaviors, respondents who engaged in any or regular physical activity, versus none, were more likely to meditate (e.g. regular physical activity, OR = 1.6, 95% CI 1.1–2.3, *p* < .014). Alcohol use was not significantly associated, but being a former smoker was (OR = 2.6, 95% CI 1.1–2.0, *p* < .05). Vegetarian diet was the only significant complementary health practice variable (OR = 2.6, 95% CI 1.7–4.1, *p* < .001). In terms of health status items, both back pain and depression were significant predictors of use as well as having one or more functional limitations (e.g. depression, OR = 1.7, 95% CI 1.2–2.2, *p* < .01). Two healthcare access variables were significant. Survey participants with public insurance, compared to being uninsured, were less likely to meditate. Those who reported 1–3, 4–9, and 10 or more visits to a conventional medical provider, versus no visits, were all more likely to practice spiritual meditation (e.g. 10+ visits, OR = 2.6, 95% CI 1.5–4.6, *p* < .01).

### Health-related reasons for practicing mindfulness meditation

This final analysis provided information on respondent reasons for exclusive use of mindfulness meditation in terms of wellness/self-care and treatment of health conditions (Table 4). In general, more respondents reported wellness and prevention (73%) as a reason to use meditation compared with treating a specific health problem (30%). Respondents reported use for stress management (92%), emotional well-being (91%), increased sense of control with health issues (65%), and to improve memory (57%) and sleep (56%). Only 28% reported use to improve immune function. Reporting use of meditation as a motivator of better health habits was less frequent. Use was noted for eating healthier (32%), and exercising more regularly (26%), while others noted use for reducing cigarette smoking (27%) and alcohol consumption (8%). The final set of items in the table provides information on mindfulness meditation practice related to use for specific health problems. The highest responses were for use of mindfulness meditation because it was self-care oriented (81%), holistic (79%), and natural (73%). The majority reported using meditation in combination with conventional medical treatments (70%), while only 13% reported receiving a recommendation to meditate from a medical doctor.

## Discussion

### Characteristics of individuals who practice meditation

As hypothesized, the characteristics of individuals who practiced meditation were found to be quite similar to those of individuals who used other complementary health practices [4–6, 64–66]. In the comparison of meditators (combined) and non-meditators (Table 1) the two groups differed predictably from each other on demographic factors, health behaviors, health status, and healthcare access. As examples, meditators were significantly more likely to be 45–64 years of age, female, White, college graduates, with higher incomes, and living in the West. They were more likely to be physically active, consume alcohol, be up-to-date for cholesterol screening, and use other complementary health practices. They also had a healthier body weight status, indicated more functional limitations, chronic low back pain, and depression, had private health insurance, and visited conventional healthcare providers more frequently in the past year. Results in Table 2 showed that across the three groups, meditation prevalence was typically higher for females, non-Hispanic Whites, college educated; respondents who were physically active, used acupuncture, yoga, vegetarian diets; and who reported depression as well as higher utilization of conventional healthcare services. The regression results showed important distinctions across the three types of meditation, such as the role of physical activity and education as predictors of spiritual meditation, while also showing several common predictors, such as having tried a vegetarian diet.

### Comparisons on key sociodemographic characteristics

One might expect practitioners of the more Eastern-rooted mindfulness and mantra meditations to be different from their Western-oriented spiritual meditation counterparts. Although the three meditations have unique histories, ontological perspectives, and practices, there was a great deal of similarity in user profiles. This similarity across numerous variables may suggest that practicing meditation is more about the type of individuals who choose to meditate than it is about the types of practices they select. One notable difference between the three methods, however, was the higher use of spiritual meditation in the sample, approximately 7 million for lifetime use in the overall sample, compared with about 4 million each for mantra and mindfulness. One contributing factor may be use of spiritual meditation as part of institutionalized mainstream American religious practice, such as use of contemplative prayer by Christians. In the United States 71% of the population identifies as Christian, whereas 6% identifies as Non-Christian, including Buddhist [70].

Given the substantial numbers of individuals practicing spiritual meditation it may be useful to increase exploration and research in this area. Indeed, a search of the PubMed database for the term “meditation” in article titles and abstracts produced over 3400 citations. A search for studies with “Zen meditation” in the title resulted in 52 articles published between 1965 and 2016; “Transcendental Meditation” produced 334 articles published between 1970 and 2016; and “Mindfulness/Vipassana meditation” produced 471 articles published between 1982 and 2016. Examining trends in publication from 2010 to 2016 showed that 17% of Transcendental Meditation studies were published during this timeframe, 37% of the Zen studies, and 75% for mindfulness meditation. Mindfulness is clearly the method currently receiving significant research attention. The resurgence in Zen research may be a consequence of it being identified as more of an open-monitoring Buddhist meditation practice, like mindfulness. A comparable search for terms related to spiritual meditation, including “centering prayer,” “contemplative prayer,” and “contemplative meditation,” produced a small number of articles mostly published within the last ten years. For example, one study compared centering prayer and mindfulness for the treatment of depression [71].

### Health behaviors

There were a number of similarities as well as important differences between the three meditation groups related to health behaviors. Alcohol use, as an example, highlights the hypothesized difference between spiritual meditation and the other two methods. In Table 1 there was no significant difference between meditators and non-meditators related to being a former drinker. In Table 2, however, when compared across the three meditations, while mindfulness and mantra were prevalent among respondents who reported moderate/infrequent alcohol consumption, spiritual meditation had the highest prevalence with “former drinker.” This may reflect use of meditation practices in support of alcohol treatment and sobriety. Alcoholics Anonymous integrates prayer and meditation into their core therapeutic model [72]. A longitudinal study of individuals with alcohol use disorders found significant increases on various spiritual/religious measures during and following treatment as well as reductions in heavy drinking [73]. Meditation has been shown to be a helpful adjunctive self-management strategy to address alcohol and substance use challenges [74–77]. In the regression analysis (Table 3), current alcohol use compared with lifetime abstinence was highly predictive of mantra and mindfulness. There was no statistical significance for spiritual meditation and current alcohol use. This may relate to the association between spiritual meditation and religiosity. The 2000 National Alcohol Survey and related studies have shown a strong relationship between conventional religiosity and alcohol abstinence [78–80].

Cigarette smoking across the three methods similarly showed spiritual meditation as having the highest prevalence with “former smoker” for smoking status (Table 2). Being a former smoker was also predictive of practicing spiritual meditation in the regression analysis, but not for the other two meditation practices. When providing reasons for use of mindfulness (Table 4), 27% of respondents who currently smoked cigarettes and 8% who currently consumed alcohol reported that meditation helped motivate them to cut back on these behaviors.

Use of vegetarian diet was also common for meditators (12%) compared with non-meditators (2%) and was a significant predictor of all three meditation practices in the regression analysis. A 2012 Gallup poll found that 5% of the US population considers themselves to be vegetarian [81]. Consumer research has found adoption of vegetarian diets to be a growing dietary choice, for both ethical and health reasons, and less likely to be adopted by individuals with more conservative cultural values [82, 83]. In a similar vein, respondents’ reasons for practicing mindfulness meditation notably included eating healthier (32%) (Table 4). Others have reported a positive benefit of mindfulness in the self-regulation of eating [84].

### Health status

There were statistically significant differences between meditators (combined) and non-meditators on the health status variables of BMI, functional limitation, back pain, depression, and number of emergency room visits. In Table 2 it was observed that among the three practices use of spiritual meditation was more prevalent among those with functional limitations; mantra and spiritual were more prevalent among those who reported back pain; and use of all three was typical for those who reported depression. It may be noteworthy that spiritual meditation, the largest meditation sample, was the only style where prevalence was higher within all three health concerns (functional limitations, back pain, depression), and in the regression analysis, the only style for which all three health concerns were significant predictors of meditation practice.

Use of any meditation in relation to health complaints may reflect a search for solutions to complex, chronic conditions. Indeed, use of complementary health practices by individuals with chronic health conditions is common [4–6]. Related research has shown use of CHPs to be related to symptom management and perceived efficacy [85]. Notably, association with lower back pain in the previous 3 months was not found to be significantly associated with either mantra or mindfulness meditation in the regression analysis but was a significant predictor for use of spiritual meditation. Chronic back pain is a condition generally noted to be one of the primary reasons for any CHP use [4, 5, 66].

Depression in the past 12 months was highly significant in relation to use of mindfulness and spiritual meditation in the regression analysis, and approached significance for mantra meditation. CHP users have been found to hold beliefs that psychological factors contribute to health and disease [86]. Such a belief could contribute to viewing meditation as a useful resource for depression and emotional well-being. Indeed, reasons for practicing mindfulness meditation reported in Table 4 included reducing stress, feeling better emotionally, and improving memory and sleep. Meditation practices, particularly mindfulness-based methods, such as MBCT, are now being integrated into mental health interventions with some evidence of benefit for depression and other mental health needs [13, 15, 87–89]. Perhaps the association of meditation with mental health may be related to growing media attention or greater service availability.

### Healthcare access

Finally, all three of the meditation practices were found to be associated with a greater utilization of conventional healthcare services. Spiritual meditation had significant associations at all three levels of conventional healthcare utilization (1–3, 4–9, 10+ visits) in regression analysis. Mindfulness had significant associations for two levels of use (1–3, 10+ visits). Higher service utilization may be related to a number of potential factors, such as actual differences in health status, personality traits, or sociodemographics. In terms of health status and specific health complaints, related work has found CHP use to be associated with having a diagnosed chronic disease, such as low back pain, and having been hospitalized in the previous 12 months [4, 5, 90]. Mantra meditation was the least significantly associated in regression analysis, and it was also the only practice for which use of acupuncture was positively associated. Perhaps individuals using mantra meditation may be more culturally identified with Eastern traditions and practices, seeking non-Western approaches such as acupuncture for health and healing. Use of mantra meditation was also not significantly associated in the regression analysis with back pain, depression, or functional limitations, another possible reason for less care-seeking behavior.

Personality traits have also been proposed as potential contributors to the use of complementary health practices [85]. In previous research CHP use was not found to be associated with the personality factor of neuroticism, but was positively associated with the personality factors of openness and agreeableness. These latter two factors were shown to relate to holistic and proactive health motives and interest in shared decision-making [86, 91].

Higher healthcare utilization could also be related to education. Of the eight demographic variables in the regression analysis, education was strongly associated with mindfulness and especially with spiritual meditation. Low educational attainment has been shown to be significantly associated with lower levels of health literacy and health knowledge [92, 93]. Limited health knowledge and lower health literacy influence the practice of preventive health behaviors, utilization of conventional healthcare services, disease prognosis and management, and expenditures on healthcare [94–98]. Higher educational attainment and higher health literacy have both been found to be positively associated with higher CHP use [99–101]. Higher CHP use has also been shown to be related to numerous preventive health practices, including moderate alcohol consumption, not smoking, low fat diet (such as a vegetarian diet), healthier body mass index, more leisure time physical activity, and use of preventive medical services, such as cholesterol screening [102–105]. Indeed, Table 2 results showed that the prevalence of all three meditation styles was higher among those who were physically active, used other complementary health practices, and had more education. Wellness and prevention was cited as a reason for use of mindfulness meditation by 73% of respondents (Table 4), paralleling the wellness-orientation found for use of yoga, another popular self-care CHP modality, in related survey studies [106, 107].

### Treatment or prevention

Respondents provided information on reasons for exclusive use of mindfulness meditation in terms of wellness/self-care and treatment of health conditions (Table 4). Mindfulness was the focus for this analysis as it is growing in use among consumers, it is currently the most actively researched meditation method, and variants of the method have been adopted into diverse secular settings. It has also been integrated into healthcare settings for physical and psychological care, such as use of mindfulness-based stress reduction (MBSR) and mindfulness-based cognitive therapy (MBCT).

In general, more respondents reported wellness and prevention (73%) as a reason to use mindfulness meditation compared with treating a specific health problem (30%), as hypothesized. Meditation for stress management and emotional well-being were commonly reported. In terms of use of meditation as a treatment, 26% indicated use because medical treatments did not work for their condition. By contrast, 70% indicated that meditation combined with medical treatment would be helpful. Other national surveys also reported that CHP use tends to be complementary, used along with conventional care, rather than used purely as an alternative [4]. Meditation was also viewed as self-care oriented (81%), holistic (79%), and natural (73%). Users of other complementary health practices cite “holistic/whole person” and “natural” as important reasons for use, as well as beliefs in the role of psychological factors in health and disease [86, 108–110]. Only 13% reported receiving a recommendation to try meditation from a medical doctor. Patient-provider communication related to CHP use remains a challenge [111]. In total these findings suggest a preventive/self-care ethos among many individuals who practice meditation.

### Strengths and limitations of the study

A major strength of this study is that the data are from a nationally representative sample of US adults, allowing for population estimates. The large sample size enables estimation of the characteristics of use of selected complementary health practices collected in the NHIS. Study limitations include the cross-sectional nature of the survey, which does not support conclusions regarding causality. Also, responses are dependent on accurate participant recall of complementary health practices and their willingness to report on their use.

## Conclusion

This paper provides a comparative view of three commonly used meditation practices—mantra, mindfulness, and spiritual meditation. Results showed that across the three practices meditators were similar to users of other popular CHPs, such as yoga. These results suggest that use of meditation may be more about the type of person practicing than about the specific type of meditation practice employed. There were also important differences observed between groups. Comparisons of exclusive practice groups found use of spiritual meditation to be more prevalent among those reporting health complaints, utilizing more conventional health services, and being a former drinker and/or former smoker, as well as constituting the largest meditation group. Review of the literature provides limited information on the use of spiritual meditation related to healthcare, mental health services, or drug and alcohol treatment. Increased research on spiritual meditation may be warranted. Finally, meditation appears to provide an accessible, self-care resource that has potential value for supporting mental health and emotional well-being, behavioral self-regulation, and integrative medical care. There is significant interest in the subject of meditation generally and mindfulness meditation specifically. Considering the nature of consumer preference for seemingly distinct types of meditation practices, understanding the underlying mechanisms, benefits, and applications of practice variations is important.

## Abbreviations

CHP: Complementary health practices
NHIS: National Health Interview Survey
NCCIH: National Center for Complementary and Integrative Health

## References

[1] Burke A, Gonzalez A (2011). Growing interest in meditation in the United States. Biofeedback.

[2] Murphy M, Donovan S, Taylor E (1997). The physical and psychological effects of meditation: a review of contemporary research.

[3] Boccia M, Piccardi L, Guariglia P (2015). The meditative mind: a comprehensive meta-analysis of MRI studies. Biomed Res Int.

[4] Barnes PM, Powell-Griner E, McFann K, Nahin RL (2004). Complementary and alternative medicine use among adults: United States, 2002. Semin Integ Med.

[5] Barnes PM, Bloom B, Nahin RL (2008). Complementary and alternative medicine use among adults and children: United States, 2007. Natl Health Stat Report.

[6] Clarke TC, Black LI, Stussman BJ, Barnes PM, Nahin RL (2015). Trends in the use of complementary health approaches among adults: United States, 2002–2012. Natl Health Stat Report..

[7] Cramer H, Hall H, Leach M, Frawley J, Zhang Y, Leung B, Adams J, Lauche R (2016). Prevalence, patterns, and predictors of meditation use among US adults: a nationally representative survey. Sci Rep.

[8] Lauricella S (2016). The ancient-turned-new concept of “spiritual hygiene”: an investigation of media coverage of meditation from 1979 to 2014. J Relig Health.

[9] Cahn BR, Polich J (2006). Meditation states and traits: EEG, ERP, and neuroimaging studies. Psychol Bull.

[10] Hölzel BK, Ott U, Gard T, Hempel H, Weygandt M, Morgen K, Vaitl D (2008). Investigation of mindfulness meditation practitioners with voxel-based morphometry. Soc Cogn Affect Neurosci.

[11] Lazar SW, Kerr CE, Wasserman RH, Gray JR, Greve DN, Treadway MT, McGarvey M, Quinn BT, Dusek JA, Benson H, Rauch SL (2005). Meditation experience is associated with increased cortical thickness. Neuroreport.

[12] Luders E, Clark K, Narr KL, Toga AW (2011). Enhanced brain connectivity in long-term meditation practitioners. NeuroImage.

[13] Chiesa A, Serretti A (2009). Mindfulness-based stress reduction for stress management in healthy people: a review and meta-analysis. J Altern Complement Med.

[14] Creswell JD, Pacilio LE, Lindsay EK, Brown KW (2014). Brief mindfulness meditation training alters psychological and neuroendocrine responses to social evaluative stress. Psychoneuroendocrinology.

[15] Goyal M, Singh S, Sibinga EM, Gould NF, Rowland-Seymour A, Sharma R, Berger Z, Sleicher D, Maron DD, Shihab HM, Ranasinghe PD (2014). Meditation programs for psychological stress and well-being: a systematic review and meta-analysis. JAMA Intern Med.

[16] Keng SL, Smoski MJ, Robins CJ (2011). Effects of mindfulness on psychological health: a review of empirical studies. Clin Psychol Review.

[17] Rubia K (2009). The neurobiology of meditation and its clinical effectiveness in psychiatric disorders. Biol Psychol.

[18] Jha AP, Krompinger J, Baime MJ (2007). Mindfulness training modifies subsystems of attention. Cogn Affect Behav Neurosci.

[19] MacLean KA, Ferrer E, Aichele SR, Bridwell DA, Zanesco AP, Jacobs TL, King BG, Rosenberg EL, Sahdra BK, Shaver PR, Wallace BA (2010). Intensive meditation training improves perceptual discrimination and sustained attention. Psychol Sci.

[20] Goldin PR, Gross JJ (2010). Effects of mindfulness-based stress reduction (MBSR) on emotion regulation in social anxiety disorder. Emotion.

[21] Lutz A, Brefczynski-Lewis J, Johnstone T, Davidson RJ (2008). Regulation of the neural circuitry of emotion by compassion meditation: effects of meditative expertise. PLoS One.

[22] Robins CJ, Keng SL, Ekblad AG, Brantley JG (2012). Effects of mindfulness-based stress reduction on emotional experience and expression: a randomized controlled trial. J Clin Psychol.

[23] Jacobs TL, Epel ES, Lin J, Blackburn EH, Wolkowitz OM, Bridwell DA, Zanesco AP, Aichele SR, Sahdra BK, MacLean KA, King BG (2011). Intensive meditation training, immune cell telomerase activity, and psychological mediators. Psychoneuroendocrinology.

[24] Kurth F, Cherbuin N, Luders E (2015). Reduced age-related degeneration of the hippocampal subiculum in long-term meditators. Psychiatry Res.

[25] Pagnoni G, Cekic M (2007). Age effects on gray matter volume and attentional performance in Zen meditation. Neurobiol Aging.

[26] Nidich S, Mjasiri S, Nidich R, Rainforth M, Grant J, Valosek L, Chang W, Zigler RL (2011). Academic achievement and transcendental meditation: a study with at-risk urban middle school students. Education.

[27] Tang YY, Tang R, Jiang C, Posner MI (2014). Short-term meditation intervention improves self-regulation and academic performance. J Child Adolesc Behav.

[28] Segal ZV, Williams JMG, Teasdale JD (2002). Mindfulness-based cognitive therapy for depression: a new approach to relapse prevention.

[29] Strauss C, Cavanagh K, Oliver A, Pettman D (2014). Mindfulness-based interventions for people diagnosed with a current episode of an anxiety or depressive disorder: a meta-analysis of randomised controlled trials. PLoS One.

[30] Wanden-Berghe RG, Sanz-Valero J, Wanden-Berghe C (2010). The application of mindfulness to eating disorders treatment: a systematic review. Eat Disord.

[31] Davidson RJ, Dunne J, Eccles JS, Engle A, Greenberg M, Jennings P, Jha A, Jinpa T, Lantieri L, Meyer D, Roeser RW (2012). Contemplative practices and mental training: prospects for American education. Child Dev Perspect.

[32] Meiklejohn J, Phillips C, Freedman ML, Griffin ML, Biegel G, Roach A, Frank J, Burke C, Pinger L, Soloway G, Isberg R (2012). Integrating mindfulness training into K-12 education: fostering the resilience of teachers and students. Mindfulness.

[33] Schonert-Reichl KA, Lawlor MS (2010). The effects of a mindfulness-based education program on pre-and early adolescents’ well-being and social and emotional competence. Mindfulness.

[34] Bazarko D, Cate RA, Azocar F, Kreitzer MJ (2013). The impact of an innovative mindfulness-based stress reduction program on the health and well-being of nurses employed in a corporate setting. J Workplace Behav Health.

[35] Chapman M. Mindfulness in the workplace: what is all the fuss about. Counselling at Work. 2011:20–4.

[36] Himelstein S (2011). Meditation research: the state of the art in correctional settings. Int J Offender Ther Comp Criminol.

[37] Stanley EA, Schaldach JM, Kiyonaga A, Jha AP (2011). Mindfulness-based mind fitness training: a case study of a high-stress predeployment military cohort. Cogn Behav Pract.

[38] Bowen S, Witkiewitz K, Dillworth TM, Chawla N, Simpson TL, Ostafin BD, Larimer ME, Blume AW, Parks GA, Marlatt GA (2006). Mindfulness meditation and substance use in an incarcerated population. Psychol Addict Behav.

[39] Brewer JA, Bowen S, Smith JT, Marlatt GA, Potenza MN (2010). Mindfulness-based treatments for co-occurring depression and substance use disorders: what can we learn from the brain?. Addiction.

[40] Gotink RA, Chu P, Busschbach JJ, Benson H, Fricchione GL, Hunink MM (2015). Standardised mindfulness-based interventions in healthcare: an overview of systematic reviews and meta-analyses of RCTs. PLoS One.

[41] Mars TS, Abbey H (2010). Mindfulness meditation practise as a healthcare intervention: a systematic review. Int J Osteopath Med.

[42] Shennan C, Payne S, Fenlon D (2011). What is the evidence for the use of mindfulness-based interventions in cancer care?. A review Psychooncology.

[43] Ospina MB, Bond K, Karkhaneh M, Buscemi N, Dryden DM, Barnes V, Carlson LE, Dusek JA, Shannahoff-Khalsa D (2008). Clinical trials of meditation practices in health care: characteristics and quality. J Altern Complement Med.

[44] Tang YY, Hölzel BK, Posner MI (2015). The neuroscience of mindfulness meditation. Nat Rev Neurosci.

[45] Awasthi B (2013). Issues and perspectives in meditation research: in search for a definition. Front Psychol.

[46] Eifring H (2015). Meditation and culture: the interplay of practice and context.

[47] Hollenback J (1996). Mysticism: experience, response, and empowerment.

[48] Nelson JM (2009). Psychology, religion, and spirituality.

[49] Bærentsen KB, Stødkilde-Jørgensen H, Sommerlund B, Hartmann T, Damsgaard-Madsen J, Fosnæs M, Green AC (2010). An investigation of brain processes supporting meditation. Cogn Process.

[50] Bishop SR, Lau M, Shapiro S, Carlson L, Anderson ND, Carmody J, Segal ZV, Abbey S, Speca M, Velting D, Devins G (2004). Mindfulness: a proposed operational definition. Clin Psychol.

[51] Bond K, Ospina MB, Hooton N, Bialy L, Dryden DM, Buscemi N, Shannahoff-Khalsa D, Dusek J, Carlson LE (2009). Defining a complex intervention: the development of demarcation criteria for “meditation”. Psycholog Relig Spiritual.

[52] Brown KW, Ryan RM (2004). Perils and promise in defining and measuring mindfulness: observations from experience. Clin Psychol.

[53] Dunn BR, Hartigan JA, Mikulas WL (1999). Concentration and mindfulness meditations: unique forms of consciousness. Appl Psychophysiol Biofeedback.

[54] Hayes SC, Shenk C (2004). Operationalizing mindfulness without unnecessary attachments. Clin Psychol.

[55] Hölzel BK, Lazar SW, Gard T, Schuman-Olivier Z, Vago DR, Ott U (2011). How does mindfulness meditation work? Proposing mechanisms of action from a conceptual and neural perspective. Perspect Psychol Sci.

[56] Lutz A, Jha AP, Dunne JD, Saron CD (2015). Investigating the phenomenological matrix of mindfulness-related practices from a neurocognitive perspective. Am Psychol.

[57] Manna A, Raffone A, Perrucci MG, Nardo D, Ferretti A, Tartaro A, Londei A, Del Gratta C, Belardinelli MO, Romani GL (2010). Neural correlates of focused attention and cognitive monitoring in meditation. Brain Res Bull.

[58] Travis F, Shear J (2010). Focused attention, open monitoring and automatic self-transcending: categories to organize meditations from Vedic. Buddhist and Chinese traditions Conscious Cogn.

[59] Stussman BJ, Bethell CD, Gray C, Nahin RL (2013). Development of the adult and child complementary medicine questionnaires fielded on the National Health Interview Survey. BMC Complement Altern Med.

[60] Monier-Williams M, Leumann E, Cappeller C (2000). A Sanskrit-English dictionary.

[61] Burke A (2012). Comparing individual preferences of four meditation techniques: zen, mindfulness, qigong, and mantra. Explore NY.

[62] Kok BE, Singer T (2017). Phenomenological fingerprints of four meditations: differential state changes in affect, mind-wandering, meta-cognition, and interoception before and after daily practice across 9 months of training. Mindfulness.

[63] National Center for Health Statistics. National Health Interview Survey. Centers for Disease Control and Prevention. 2012. www.cdc.gov/nchs/nhis.htm. Accessed Feb 2015.

[64] Nahin RL, Dahlhamer JM, Stussman BJ (2010). Health need and the use of alternative medicine among adults who do not use conventional medicine. BMC Health Serv Res.

[65] Schoenborn CA, Adams PE. Health behaviors of adults: United States, 2005–2007. Vital Health Stat 10. 2010;245:1–132. 20669609

[66] Nahin RL, Boineau R, Khalsa PS, Stussman BJ, Weber WJ (2016). Evidence-based evaluation of complementary health approaches for pain management in the United States. Mayo Clin Proc.

[67] Burke A, Nahin RL, Stussman BJ (2015). Limited health knowledge as a reason for non-use of four common complementary health practices. PLoS One.

[68] Parsons VL, Moriarity C, Jonas K, Moore TF, Davis KE, Tompkins L. Design and estimation for the national health interview survey, 2006–2015. Vital Health Stat 2. 2014;165:1–53. 24775908

[69] StataCorp. Stata Statistical Software: Release 13. College Station: StataCorp; 2013.

[70] Smith G, Cooperman A, Mohamed B, Martinez J, Alper B, Sciupac E, Ochoa J (2015). America’s changing religious landscape.

[71] Knabb JJ (2012). Centering prayer as an alternative to mindfulness-based cognitive therapy for depression relapse prevention. J Relig Health.

[72] Forcehimes A (2004). De profundis: spiritual transformations in alcoholics anonymous. J Clin Psychol.

[73] Robinson EA, Cranford JA, Webb JR, Brower KJ (2007). Six-month changes in spirituality, religiousness, and heavy drinking in a treatment-seeking sample. J Stud Alcohol Drugs.

[74] Delaney HD, Forcehimes AA, Campbell WP, Smith BW (2009). Integrating spirituality into alcohol treatment. J Clin Psychol.

[75] Khusid MA, Vythilingam M (2016). The emerging role of mindfulness meditation as effective self-management strategy, part 2: clinical implications for chronic pain, substance misuse, and insomnia. Mil Med.

[76] Witkiewitz K, Marlatt GA, Walker D (2005). Mindfulness-based relapse prevention for alcohol and substance use disorders. J Cogn Psychother.

[77] Zgierska A, Rabago D, Chawla N, Kushner K, Koehler R, Marlatt A (2009). Mindfulness meditation for substance use disorders: a systematic review. Subst Abus.

[78] Rogers RG, Krueger PM, Miech R, Lawrence EM, Kemp R (2013). Nondrinker mortality risk in the United States. Popul Res Policy Review.

[79] Michalak L, Trocki K, Bond J (2007). Religion and alcohol in the US National Alcohol Survey: how important is religion for abstention and drinking. Drug Alcohol Depend.

[80] Wallace JM, Brown TN, Bachman JG, Laveist TA (2003). The influence of race and religion on abstinence from alcohol, cigarettes and marijuana among adolescents. J Stud Alcohol.

[81] Newport F. In US, 5% consider themselves vegetarians. Gallup Wellbeing. 2012.

[82] Dietz T, Frisch AS, Kalof L, Stern PC, Guagnano GA (1995). Values and vegetarianism: an exploratory analysis. Rural Sociol.

[83] Jabs J, Devine CM, Sobal J (1998). Model of the process of adopting vegetarian diets: health vegetarians and ethical vegetarians. J Nutr Educ Behav.

[84] Mason AE, Epel ES, Aschbacher K, Lustig RH, Acree M, Kristeller J, Cohn M, Dallman M, Moran PJ, Bacchetti P, Laraia B (2016). Reduced reward-driven eating accounts for the impact of a mindfulness-based diet and exercise intervention on weight loss: data from the SHINE randomized controlled trial. Appetite.

[85] Astin JA (1998). Why patients use alternative medicine: results of a national study. JAMA.

[86] Furnham A (2007). Are modern health worries, personality and attitudes to science associated with the use of complementary and alternative medicine?. Br J Health Psychol.

[87] Hofmann SG, Sawyer AT, Witt AA, Oh D (2010). The effect of mindfulness-based therapy on anxiety and depression: a meta-analytic review. J Consult Clin Psychol.

[88] Khoury B, Sharma M, Rush SE, Fournier C (2015). Mindfulness-based stress reduction for healthy individuals: a meta-analysis. J Psychosom Res.

[89] Khusid MA, Vythilingam M (2016). The emerging role of mindfulness meditation as effective self-management strategy, part 1: clinical implications for depression, post-traumatic stress disorder, and anxiety. Mil Med.

[90] Saydah SH, Eberhardt MS (2006). Use of complementary and alternative medicine among adults with chronic diseases: United States 2002. J Altern Complement Med.

[91] Sirois FM, Purc-Stephenson RJ (2008). Personality and consultations with complementary and alternative medicine practitioners: a five-factor model investigation of the degree of use and motives. J Altern Complement Med.

[92] Kutner M, Greenberg E, Jin Y, Paulsen C (2006). The health literacy of America’s adults: results from the 2003 National Assessment of adult literacy (NCES 2006–483).

[93] Martin LT, Ruder T, Escarce JJ, Ghosh-Dastidar B, Sherman D, Elliott M, Bird CE, Fremont A, Gasper C, Culbert A, Lurie N (2009). Developing predictive models of health literacy. J Gen Intern Med.

[94] Anderson R, Newman JF (1973). Societal and individual determinants of medical care utilization in the United States. Milbank Mem Fund Q Health Soc.

[95] Berkman ND, Sheridan SL, Donahue KE, Halpern DJ, Crotty K (2011). Low health literacy and health outcomes: an updated systematic review. Ann Intern Med.

[96] Macabasco-O’Connell A, DeWalt DA, Broucksou KA, Hawk V, Baker DW, Schillinger D, Ruo B, Bibbins-Domingo K, Holmes GM, Erman B, Weinberger M (2011). Relationship between literacy, knowledge, self-care behaviors, and heart failure-related quality of life among patients with heart failure. J Gen Intern Med.

[97] Schillinger D, Grumbach K, Piette J, Wang F, Osmond D, Daher C, Palacios J, Sullivan GD, Bindman AB (2002). Association of health literacy with diabetes outcomes. JAMA.

[98] Wolf MS, Gazmararian JA, Baker DW (2005). Health literacy and functional health status among older adults. Arch Intern Med.

[99] Bains SS, Egede LE (2011). Association of health literacy with complementary and alternative medicine use: a cross-sectional study in adult primary care patients. BMC Complement Altern Med.

[100] Cui Y, Hargreaves MK, Shu XO, Liu J, Kenerson DM, Signorello LB, Blot WJ (2012). Prevalence and correlates of complementary and alternative medicine services use in low-income African Americans and whites: a report from the southern community cohort study. J Altern Complement Med.

[101] Gardiner P, Mitchell S, Filippelli AC, Sadikova E, White LF, Paasche-Orlow MK, Jack BW (2013). Health literacy and complementary and alternative medicine use among underserved inpatients in a safety net hospital. J Health Commun.

[102] Druss BG, Rosenheck RA (1999). Association between use of unconventional therapies and conventional medical services. JAMA.

[103] Gray CM, Tan AW, Pronk NP, O'Connor PJ (2002). Complementary and alternative medicine use among health plan members: a cross-sectional survey. Eff Clin Pract.

[104] Nahin RL, Dahlhamer JM, Taylor BL, Barnes PM, Stussman BJ, Simile CM, Blackman MR, Chesney MA, Jackson M, Miller H, McFann KK (2007). Health behaviors and risk factors in those who use complementary and alternative medicine. BMC Public Health.

[105] Robinson AR, Crane LA, Davidson AJ, Steiner JF (2002). Association between use of complementary/alternative medicine and health-related behaviors among health fair participants. Prev Med.

[106] Saper HB, Eisenberg DM, Davis RB, Culpepper L, Phillips RS (2004). Prevalence and patterns of adult yoga use in the United States: results of a national survey. Altern Ther Health Med.

[107] Stussman BJ, Black LI, Barnes PM, Clarke TC, Nahin RL (2015). Wellness-related use of common complementary health approaches among adults: United States, 2012. Natl Health Stat Report..

[108] Barrett B, Marchand L, Scheder J, Plane MB, Maberry R, Appelbaum D, Rakel D, Rabago D (2003). Themes of holism, empowerment, access, and legitimacy define complementary, alternative, and integrative medicine in relation to conventional biomedicine. J Altern Complement Med.

[109] Goldstein MS (1999). Alternative health care: medicine, miracle or mirage?.

[110] Schuster TL, Dobson M, Jauregui M, Blanks RH (2004). Wellness lifestyles I: a theoretical framework linking wellness, health lifestyles, and complementary and alternative medicine. J Altern Complement Med.

[111] Perlman AI, Eisenberg DM, Panush RS (1999). Talking with patients about alternative and complementary medicine. Rheum Dis Clin N Am.

